# Regulatory network of ginsenoside biosynthesis under Ro stress in the hairy roots of *Panax ginseng* revealed by RNA sequencing

**DOI:** 10.3389/fbioe.2022.1006386

**Published:** 2022-10-31

**Authors:** Xiangru Meng, Tao Zhang, Changbao Chen, Qiong Li, Jingwan Liu

**Affiliations:** Key Laboratory of Chinese Medicine Planting and Development, Changchun University of Chinese Medicine, Changchun, Jilin, China

**Keywords:** *P. ginseng*, Ro stress, physiological characteristic, ginsenoside, miRNA

## Abstract

*P. ginseng* C.A. Meyer is a valuable Chinese herbal medicine that belongs to the Araliaceae family. Major obstacles to the continuous cropping of ginseng have severely restricted the sustainable development of the ginseng industry. The allelopathic effects of triterpenoid saponins play an important role in disorders related to continuous cropping; however, the mechanisms underlying the allelopathic autotoxicity of triterpenoid ginsenosides remain unknown. In this study, we performed mRNA and miRNA sequencing analyses to identify candidate genes and miRNAs that respond differentially to ginsenoside Ro stress in ginseng and their targets. The growth of the ginseng hairy roots was significantly inhibited under Ro stress (0.5 mg/L, Ro-0.5). The inhibition of root growth and injury to root-tip cells promoted the accumulation of the endogenous hormones indole-3-acetic acid and salicylic acid and inhibited the accumulation of abscisic acid and jasmonate acid. The accumulation of ginsenosides, except Rg3, was significantly inhibited under Ro-0.5 stress. An mRNA analysis of the Ro-0.5 and control groups showed that differentially expressed genes were mostly concentrated in the hormone signal transduction pathway. ARF7 and EFM were upregulated, whereas XTH23 and ZOX1 were downregulated. These genes represent important potential candidates for hormone-responsive continuous cropping diseases. In total, 74 differentially expressed miRNAs were identified based on the miRNA sequencing analysis, of which 22 were upregulated and 52 were downregulated. The target genes of ptc-miR156k_L + 1, mtr-miR156b-5p, gma-miR156a_R + 1, and mtr-miR156e all belonged to TRINITY_DN14567_c0_g4, which is a gene in the plant hormone signal transduction pathway. These four miRNAs were all negatively correlated with mRNA, indicating their likely involvement in the response of ginseng to continuous cropping disorders and the regulation of ginsenoside synthesis. Our findings provide useful insights for removing the barriers to continuous ginseng cropping and have important implications in the genetic engineering of plant stress responses.

## Introduction

*P. ginseng* C.A. Meyer is a perennial herb and valuable traditional Chinese medicinal ingredient with a long history of use. Ginsenosides, which are the major active compounds in ginseng, have numerous medicinal benefits such as immune regulation, anti-stress, anti-fatigue, anti-oxidation, anti-inflammatory, anti-tumor, hypoglycemic, and liver-protective properties (China Pharmacopoeia Commission, 2020; Fan et al., 2019; Riaz M. et al., 2019). Approximately 200 types of ginsenoside monomers have been isolated from ginseng (Lee et al., 2012; Shi et al., 2013). Ginsenosides are critical biomarkers of ginseng quality. The 2020 edition of the Chinese Pharmacopoeia has defined the appropriate amounts of root ginsenosides for therapeutic use. For example, the amount of Rg1 (C_42_H_72_O_14_) and Re (C_48_H_82_O_18_) should not be lower than 0.30%, and that of Rb1 (C_54_H_92_O_23_) should not be lower than 0.20% (China Pharmacopoeia Commission, 2020; Sun et al., 2011). The different aglycones of ginsenosides may be divided into oleanane-type pentacyclic triterpene saponins, protopanaxadiol-type saponins (PPD), and protopanaxatriol-type saponins (PPT), where PPD- and PPT-type saponins are dammarane-type tetracyclic triterpenoids (Zhang et al., 2019).

Ginseng has the highest output value of all Chinese herbal medicines and is an important resource with unique characteristics within the Chinese medicine and general health industries. However, the cultivation period of ginseng is long, and obstacles to the continuous cropping of ginseng have long acted as a major technical bottleneck restricting its sustainable development. Soils in which ginseng has previously been planted cannot be used for ginseng production for the next 30 years because of continuous cropping issues such as burning beard, red skin, and rotten roots, which can lead to a reduced yield or no harvest of the crop. Therefore, to ensure continuous planting, China has cut down forests to plant new ginseng crops. This has resulted in serious problems such as ecological imbalance, soil erosion, and the depletion of rare forest species (Li et al., 2022). Rhizosphere allelopathy is one of the main factors inhibiting the continuous cropping of ginseng medicinal plants as it induces rhizosphere soil acidification, rhizosphere microbial community structure imbalance, and plant diseases. Moreover, the obstacles to continuous cropping have intensified over time (Li et al., 2022). Allelopathy refers to the phenomenon in which plants or microorganisms release chemical substances, or allelochemicals, into the surrounding environment, which then affect the growth and development of surrounding plants. These allelochemicals can exert positive or negative effects on plants, such as autotoxicity, partiality, and self-promotion. Allelopathy mainly occurs in the soil through root exudation, residue decomposition, and rainwater leaching, and directly or indirectly affects the formation of soil structure and plant growth. The secondary metabolites of ginseng biosynthesis are allelopathic substances and are mainly produced by the acetate or shikimate pathways or a combination of both. Allelochemicals mainly originate from root exudation and residue decomposition in the soil during plant growth. Since these components are insoluble in water, they accumulate gradually and may eventually reach a concentration at which they can seriously inhibit plant growth and development. Allelochemicals have significant inhibitory effects on *P. ginseng* and affect seed germination, seedling growth, and root vigor. A study by Chen found that the soil extract of old ginseng could inhibit ginseng seed germination and root elongation (Chen et al., 2006). Moreover, the degree of the inhibition of ginseng seed germination and root elongation increased with increasing extract concentrations. Plant hormones play an important role in the adaptation of ginseng to external environmental stress. The allelochemical ginsenoside Rg1 influences the low promotion and high inhibition of the endogenous hormone levels of ginseng seedlings; that is, low concentrations of Rg1 promote auxin [indole-3-acetic acid (IAA)] and gibberellin contents, whereas high concentrations of Rg1 reduce IAA and gibberellin contents. Furthermore, different concentrations of Rg1 may increase the amount of abscisic acid (Qiong, 2020).

mRNA and miRNA sequencing are key modern biotechnologies and important methods for exploring and mining the key genes in plants that respond to external environmental stimuli. Wu et al. (2012) constructed a miRNA transcriptome database of ginseng roots, stems, leaves, and flowers using high-throughput sequencing technology and obtained a large number of miRNAs related to environmental stress. Gao et al. (2016) used RNA sequencing to study ginseng transcriptome products treated with rust fungus at different timepoints and found that 3,839 of the 73,335 single genes that had been produced were upregulated. Chen et al. (2020) further used methyl jasmonate to stress the adventitious roots of ginseng and reported 136 upregulated PgERF genes and 152 downregulated PgERF genes. Moreover, two POD genes and one SOD gene were upregulated in ginseng root tissue, one SOD gene was upregulated in ginseng leaf tissue, and seven genes related to ginsenoside synthesis were downregulated.

In this study, we simulated the phenomenon of continuous cropping obstacles in ginseng and detected morphological changes in the hairy roots of ginseng by adding ginsenoside Ro exogenously under relatively controllable experimental conditions. We measured the degree of damage to tip cells and the contents of ginsenosides and endogenous hormones in the hairy roots of ginseng. We further explored the effects of ginsenoside Ro stress on ginsenoside biosynthesis and observed ginsenoside-induced allelopathic damage to ginseng endogenous substance accumulation. By combining mRNA and miRNA sequencing technologies, we comprehensively analyzed the toxic mechanisms of ginseng allelopathic interference at the transcriptional and post-transcriptional levels under ginsenoside Ro allelopathic stress. This study provides a theoretical basis for ensuring the continuous and effective supply of land for ginseng production.

## Results

### Biomass and morphological changes in ginseng hairy roots

**FIGURE 1:**
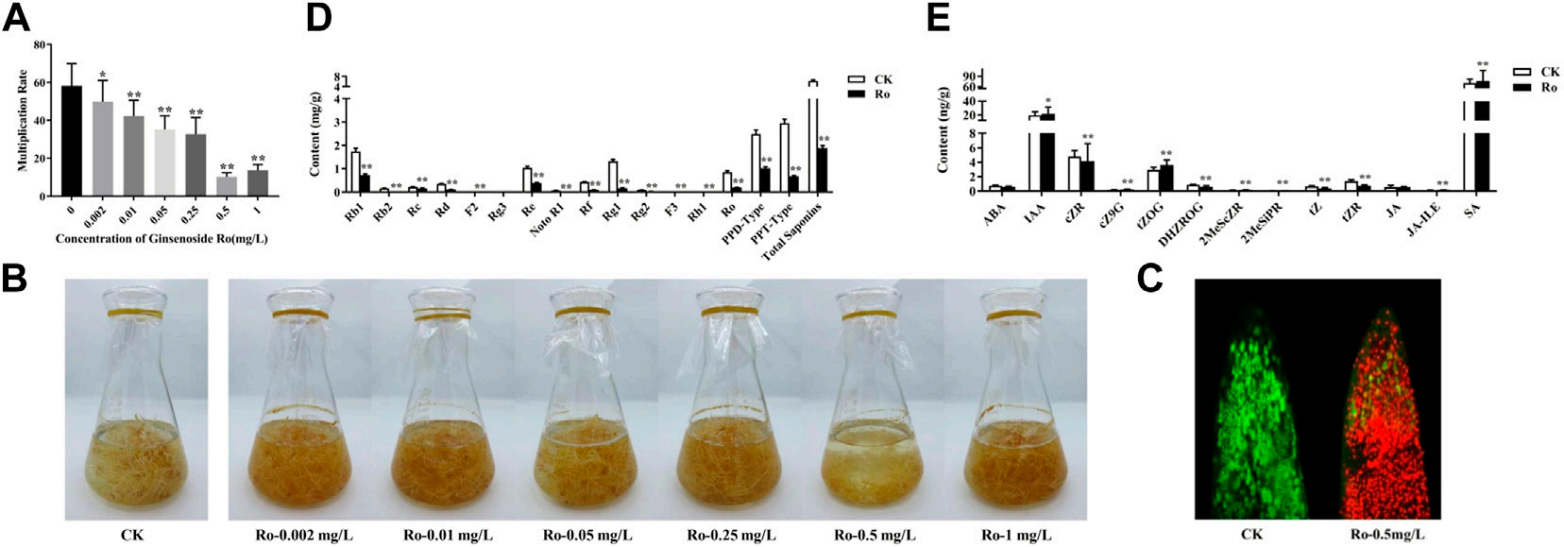
Effects of Ro on the appearance, ginsenoside content, and hormone content of the hairy roots of *P. ginseng*. Monthly multiplication rate of *P. ginseng* hairy roots induced by different concentrations of ginsenoside Ro **(A)**. Effects of different Ro concentrations on the appearance of ginseng hairy roots **(B)**. Ro-induced root-tip cell viability differences in ginseng hairy-root injury **(C)**. Changes in the content of ginsenosides in the hairy roots of *P. ginseng* **(D)**. Absolute quantitative results of 13 hormones in the hairy roots of *P. ginseng* **(E)**. * indicates a significant correlation (*p* < 0.05); ** indicates a significant correlation (*p* < 0.01).

The general growth cycle of the ginseng hairy roots lasted 30 days, with a slow growth period from days 1–13 and a logarithmic growth period from days 14–22, during which the metabolic rate was vigorous and growth rate was the highest. The ginseng entered a period of relatively stable growth after 22 days, with no further increases in weight. The final concentrations of saponin Ro in the liquid media of the six treatment groups were 0.002 mg/L (Ro 0.002), 0.01 mg/L (Ro 0.01), 0.05 mg/L (Ro 0.05), 0.25 mg/L (Ro 0.25), 0.50 mg/L (Ro 0.5), and 1.00 mg/L (Ro 1). Distilled water was used for the control group. The hairy roots of ginseng that exhibited good growth were subcultured into conical flasks for the different treatments. The initial weight of each bottle was 3 g, and the samples were weighed after 30 days. The results are shown in Figure 1A. The growth of the ginseng roots was significantly inhibited by treatment with 0.5 mg/L of Ro. The lowest monthly multiplication rate was 10.23 (Ro 0.5), which was lower than that of the CK group (82.41%). The difference was highly significant. The Ro 0.5 treatment had the greatest inhibitory effect on the growth of the ginseng hairy roots.

The growth states of the ginseng hairy roots in liquid media with different Ro concentrations differed as shown in Figure 1B. The hairy roots in the CK group were in good condition overall, with a light-yellow color, multiple hairs, multiple branches, rapid growth, and clumps. Moreover, the hairy roots were longer and thicker than those in the treatment groups with and had many fine branches. Compared to those in the CK group, the hairy roots in the treatment groups were brown in color. The growth of the hairy roots in the Ro 0.5 group showed an apoptotic trend, and their color gradually changed from fresh translucent white to dark yellow before becoming brown and dark brown after 27 days. This indicated that Ro 0.5 had a significant inhibitory effect on the growth of the ginseng hairy roots.

### Root-tip cell viability assay results

According to the measured monthly ginseng hairy-root growth rate, the Ro 0.5 treatment group exhibited the greatest inhibitory effect on hairy-root growth; therefore, this group was selected as the treatment group for further study. Confocal laser scanning microscopy was used to measure the viability of apical cells in the hairy roots, observe the ratio of living-to-dead cells, and complete the localization of apical cells in the hairy roots. Fluorescein diacetate can rapidly enter living root-tip cells and show strong fluorescence, whereas propidium iodide can rapidly enter dead root-tip cells and display strong fluorescence. The results of the co-staining experiment on the tips of the hairy root in the CK and treatment groups are shown in Figure 1C. The root cells in the CK group exhibited strong vitality and were in good condition. Conversely, the cells at the root tips in the treatment group were severely damaged. It is possible that a large amount of propidium iodide entered the dead cells, combined with DNA, and was repelled by the living cells, thereby resulting in strong red fluorescence. Therefore, red blood cells were more abundant in the treatment group than in the CK group. These results also confirmed the significant inhibitory effect of Ro 0.5 on the growth of ginseng hairy-root cells. The CK and Ro 0.5 groups were further selected for an in-depth omics sequencing analysis.

### Ginsenoside contents in ginseng hairy roots

After the exogenous addition of 0.5 mg/L of ginsenoside Ro, the accumulation of ginsenosides (except for Rg3) was significantly inhibited compared to that in the CK group. The results are shown in Figure 1D, which shows that PPD-type saponins decreased by 1.4560 mg/g to 0.59-times less than that in the CK group (*p* < 0.01); PPT-type saponins decreased by 2.2840 mg/g to 0.77-times less than that in the CK group (*p* < 0.01); and OLE-type saponins decreased by 0.6580 mg/g to 0.77-times less than that in the CK group (*p* < 0.01). Moreover, the sum of all 14 saponins decreased by 4.3980 mg/g to 0.71-times less than that in the CK group (*p* < 0.01).

After the Ro 0.5 treatment, the ratio of Rb1, Re, Rg1, PPD-type, and PPT-type ginsenosides-to-total saponins also changed (Table 1). The proportion of Rb1, Re, and PPT-type ginsenosides decreased significantly, whereas the proportion of Rg1 and PPD-type ginsenosides and that of PPD/PPT increased significantly. This indicated that the accumulation of PPD-type ginsenosides was stimulated under the Ro 0.5 treatment, whereas the biosynthesis of PPT-type ginsenosides was inhibited.

**TABLE 1:** Changes in the ratio of ginsenosides in hairy roots after Ro treatment.

Ginsenoside type	CK mean ± SD (%)	Ro 0.5 mg/L mean ± SD (%)
Rb1/Total saponins	27.64% ± 2.23%	38.52% ± 2.39%
Re/Total saponins	16.47% ± 1.10%	20.41% ± 1.16%
Rg1/Total saponins	21.05% ± 1.14%	8.67% ± 0.55%
PPD/Total saponins	39.49% ± 2.89%	54.42% ± 3.32%
PPT/Total saponins	46.98% ± 2.71%	35.39% ± 2.11%
PPD/PPT	84.06% ± 6.16%	153.78% ± 9.39%

### Endogenous hormone contents in ginseng hairy roots

Changes in the endogenous hormone contents of the ginseng hairy roots under Ro stress are shown in Figure 1E. Adding ginsenoside Ro exogenously significantly promoted the accumulation of the endogenous hormones IAA and salicylic acid (SA), which increased by 10.0% and 9.6%, respectively, compared with those in the CK group. The accumulation of abscisic acid (ABA) and its synthetic pathway intermediates was inhibited, as was the accumulation of the endogenous hormone jasmonate acid (JA) and its synthetic pathway intermediates. We speculate that the hairy roots of ginseng resist external environmental stimuli by regulating the biosynthesis of endogenous hormones. IAA, SA, JA, and ABA, in particular, are important endogenous signaling molecules in hairy ginseng roots that respond to ginsenoside Ro stress.

### Basic information on miRNA sequencing

**FIGURE 2:**
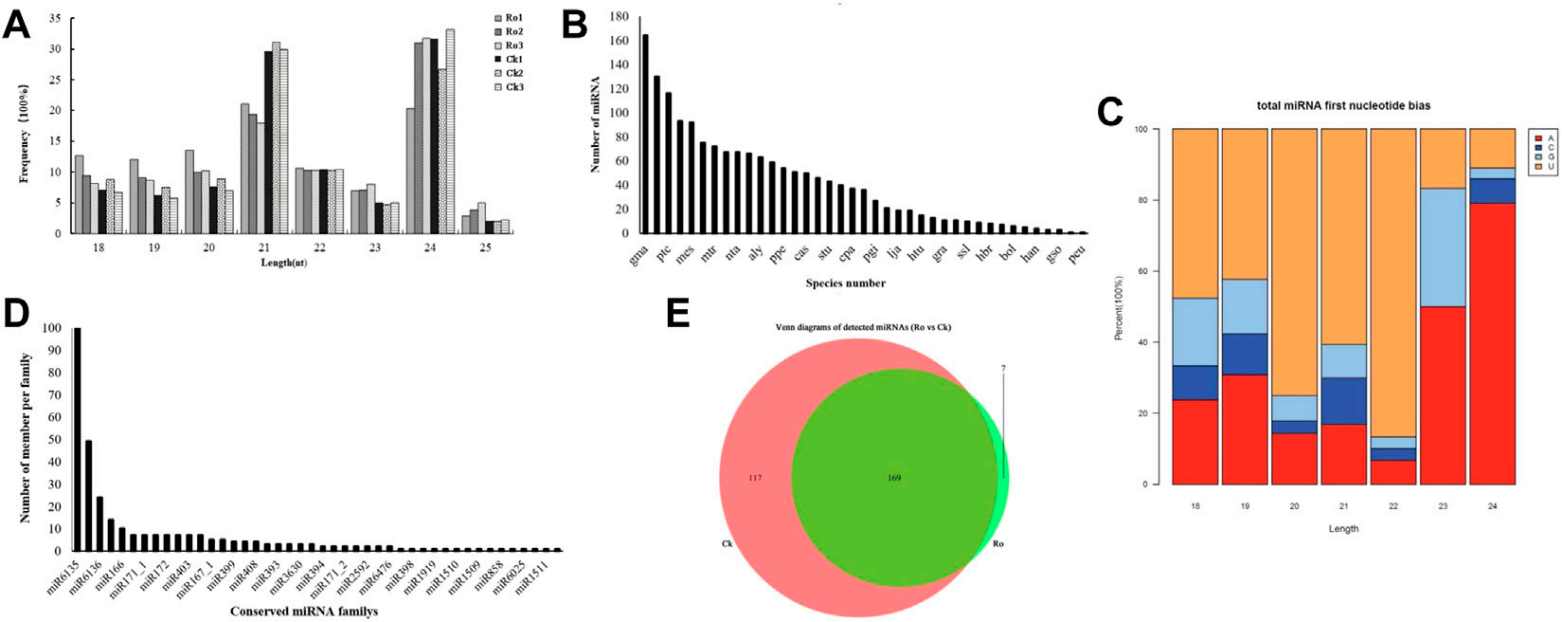
Changes in miRNAs in the hairy roots of *P. ginseng* under ginsenoside Ro stress. Length distribution of the total valid reads **(A)**. Statistics on the frequency of miRNA of *P. ginseng* in other species **(B)**. miRNA first nucleotide bias of *P. ginseng* **(C)**. Distribution of the conserved miRNAs of *P. ginseng* **(D)**. Venn diagram of the number of common and unique miRNAs in ginseng under ginsenoside Ro stress **(E)**.

Statistical and quality controls were performed on the unique sequences of the six detection samples and the copy number corresponding to each sequence. The raw sequencing data were first filtered to remove low-quality bases (<18 nt) at the linker sequence and 3′ ends of the sequences. An overview of the sequencing data and sequence alignments of the six samples (Supplementary Table S1) indicated that the ginseng RNA was of high quality. We then compared and filtered quality-controlled raw data with databases such as RFam and Repbase to obtain valid data. The numbers of valid reads in the six samples were 25,64,382, 35,87,700, 27,58,087, 36,22,380, 36,78,294, and 38,90,141. As shown in Figure 2A, the distribution of the sequence lengths of the six samples was mainly concentrated at 20–24 nt, with 21 and 24 nt being the most common lengths, which was consistent with the typical characteristics of Dicer enzyme cleavage.

miRNAs are highly evolutionarily conserved across many species. Therefore, we speculate that miRNAs identified from the same species may also be evolutionarily conserved. We counted the miRNAs that were detected in ginseng and other species (Figure 2B). Many of the conserved miRNAs in the ginseng were also highly conserved in the evolutionary process and were similar to those in soybean (*glycine*). max (*Linn*. Merr.), apples (*Malus domestica*), poplar (*Populus* L.), *Arabidopsis thaliana* (L. Heynh.), and other species, with high homology.

Regarding the sequence-specificity determinants of plant miRNAs, the first base at the 5′ end has a U bias. Therefore, we analyzed the preference of the 5′ first base of miRNAs in ginseng (Figure 2C). The sequencing results showed that the first bases of the miRNA sequences with lengths of 18–22 nt in ginseng were more inclined to U, whereas the miRNAs with lengths of 23 or 24 nt had a preference for the first base. This was consistent with previous findings on plant miRNA characterization, which enhances our confidence in our identification results (Chen, 2017).

### Identification of miRNAs in the hairy roots of ginseng under Ro stress

Next, we identified the types of known miRNAs in ginseng and their families by aligning the obtained miRNA sequences with known miRNA sequences from different species (Figure 2D). We obtained a total of 397 ginseng candidate miRNAs from 44 families. Because the miRNAs detected by the sequencing analysis of the experimental data were predominantly in the form of isoform population expression, the expression of many types of miRNAs in the sequence expression in certain stages and periods was not necessarily the same as that in the miRBase database. The reported miRNA sequences were identical. Among these miRNA families, miR6135 was the largest family and contained 185 miRNAs, followed by the miR6140 family, which contained 40 miRNAs, and the miR166 family, which contained 10 miRNAs. These results were similar to those of previous studies (Jung et al., 2018).

The miRNA Venn diagram is shown in Figure 2E. Based on the principle that the expression level of mature miRNAs in the six samples was not zero, 293 miRNAs were expressed in the samples from the same group. Moreover, 169 miRNAs were expressed in all the samples of the group. Among them, seven miRNAs were specifically expressed in the Ro group and 117 miRNAs were expressed in the CK group.

**FIGURE 3:**
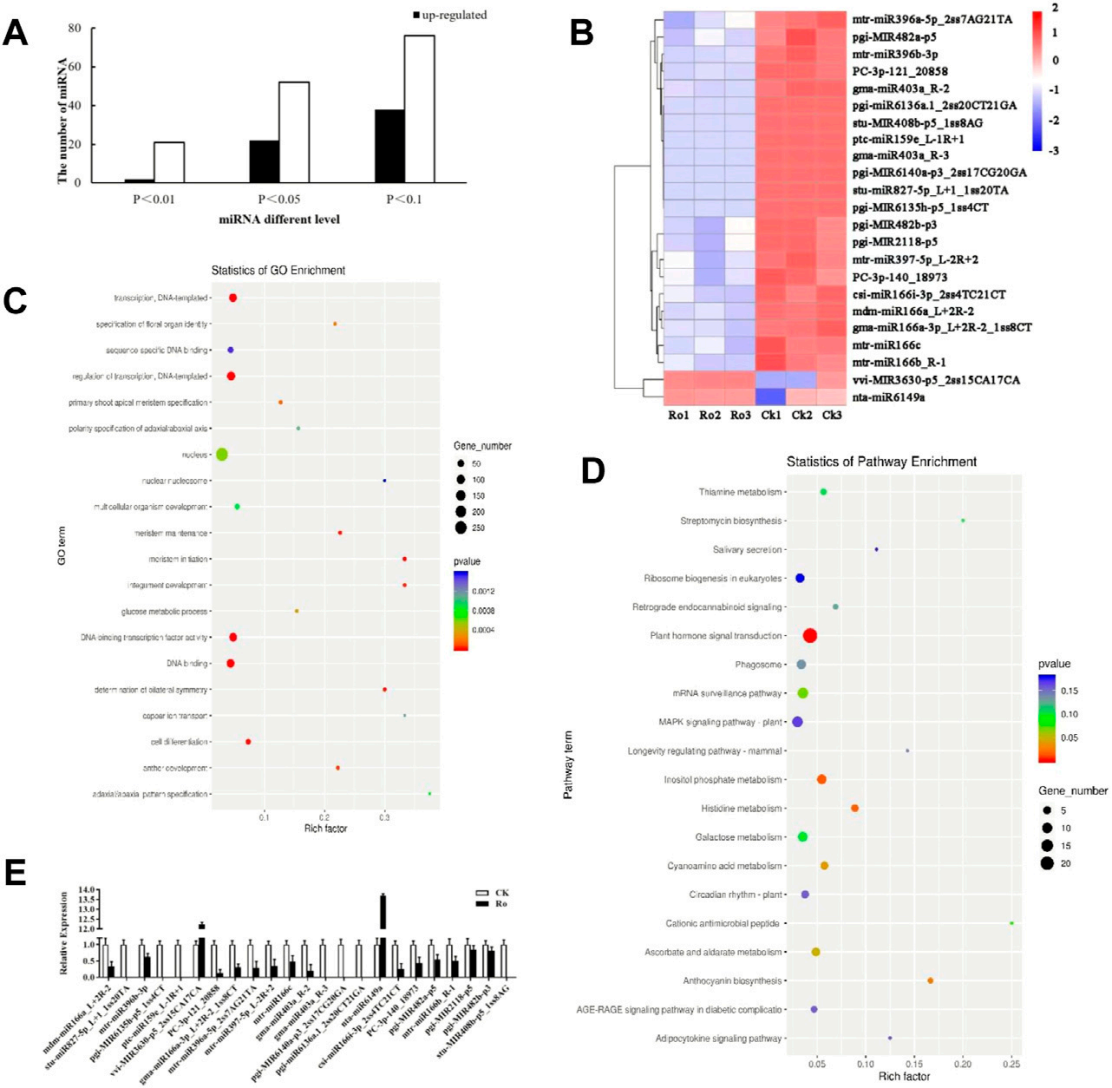
Results and validation of the differential miRNA analysis. Number of the differentially expressed miRNAs of *P. ginseng* hairy roots **(A)**. Heatmap of the differentially expressed miRNAs of *P. ginseng* hairy roots **(B)**. GO functional enrichment analysis of miRNA target genes in ginseng under Ro stress **(C)**. KEGG functional enrichment analysis of miRNA target genes in ginseng under Ro stress **(D)**. Verification of the fluorescence and quantitative PCR results of miRNA **(E)**.

Differentially expressed miRNAs were analyzed, and the number of reads obtained by sequencing was normalized to obtain the miRNA expression levels. Differentially expressed miRNAs were screened using a threshold of *p* ≤ 0.05. A total of 74 differentially expressed miRNAs were detected, of which 22 were upregulated and 52 were downregulated (Figures 3A,B). The expression levels of peu-MIR2916-p5_1ss5AG in the upregulated miRNAs and mdm-miR166a_L+2R-2, mtr-miR166c, and mtr-miR396b-5p_1ss7AG in the downregulated miRNAs were higher, which may have played an important role in the plant response to ginsenoside Ro stress.

### Target gene prediction and analysis of differential miRNAs

TargetFinder (Dai et al., 2018) was used to predict the target genes of the miRNAs with significant differences. The functions in which the predicted target genes were enriched in were analyzed using Gene Ontology (GO) and the Kyoto Encyclopedia of Genes and Genomes (KEGG). The results of the GO functional annotation analysis of the miRNA target genes are shown in Figure 3C. The target genes were mainly involved in the auxin-activated signaling pathway, multicellular organism development, cell differentiation, meristem initiation, and other processes, which indicated that the miRNAs annotated to the target genes may be involved in or correspond to cell differentiation, hormone levels, and other processes. The results of the KEGG functional annotation analysis of the miRNA target genes are shown in Figure 3D. The target genes were mainly involved in plant hormone signal transduction, terpenoid backbone biosynthesis, and circadian rhythm-plant. In the following sections of this paper, we focus on the hormone synthesis pathway. The miRNA sequencing results were verified by a real-time polymerase chain reaction (RT-PCR) analysis, and the 23 differentially expressed miRNAs were subjected to quantitative fluorescence testing. The miRNA expression patterns were consistent with the sequencing results (Figure 3E), but the sequencing results were more accurate.

### Transcriptome sequencing of ginseng hairy roots

The RNA quality results for the ginseng hairy roots are shown in Supplementary Table S1. The total mass of the six samples was greater than 2 μg, and the OD 260/280 was between 1.96 and 2.24. The Agilent Bioanalyzer 2100 was used to analyze the total RNA of the six ginseng hairy root samples. In the quantitative detection, the RNA integrity numbers (RINs) were all >7, which indicated that the integrity of the RNA met the standard and requirements needed for subsequent experiments. The sequencing data has been uploaded to the NCBI database and may be found using the following link (https://www.ncbi.nlm.nih.gov/geo/query/acc.cgi?acc=GSE212097) and accession number GSE212097.

Six cDNA libraries that had been prepared from the ginseng hairy roots were sequenced using an Illumina HiSeq 4000 platform. In total, 83,77,40,362 raw reads were generated (Supplementary Table S2). After removing joint, duplicate, and low-quality reads, the Ro (Ro1, Ro2, and Ro3) and CK (CK1, CK2, and CK3) groups comprised a total of 63,72,41,134 reads. The clean reads were determined to be those with Q20 values greater than 98%, Q30 values greater than 93%, and a GC content between 44.19 and 49.33%.

**FIGURE 4:**
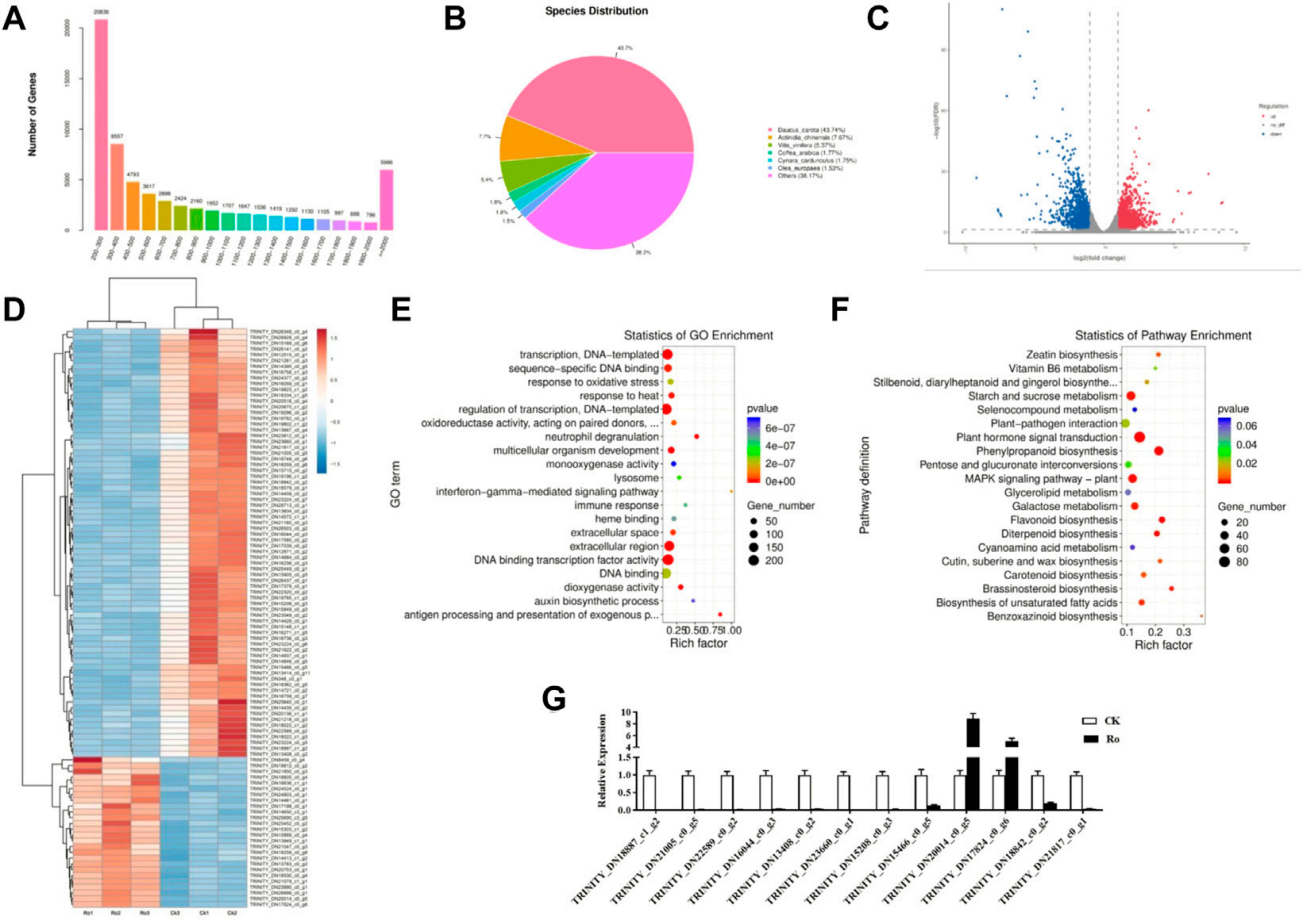
Differential mRNA analysis results and validation. Distribution of unigene lengths in the ginseng callus transcriptome; *X*, length of the unigene; *Y*, number of unigenes **(A)**. Non-redundant homologous species distribution **(B)**. Comparison of the differential genes between the Ro and CK treatments **(C)**. Hierarchical analysis of the differentially expressed genes (DEGs) **(D)**. GO enrichment analysis of the DEGs **(E)**. KEGG enrichment analysis of the DEGs **(F)**. Comparison of the RNA-seq and qPCR results **(G)**.

Because the reference genome of ginseng has many repetitive sequences, it is difficult to compare and complete its genome sequence. We therefore used an unparalleled ginseng transcriptome for the comparative analysis. Trinity software was used for the *de novo* assembly of all the clean reads of the six libraries into contigs. The reads were reflected contigs, redundancy was removed, and the longest transcript was defined as the unigene. In total, 65,730 unigenes were obtained with an N50 length of 1,404 nt. The sequencing and assembly results are shown in Supplementary Table S3, and the length distributions of all the unigenes are shown in Figure 4A. Most of the unigenes were 200–300 nt in length. As the length of the unigenes increased, their number decreased, except when they were longer than 2,000 nt.

DIAMOND software was used to obtain functional annotations, and the non-redundant, GO, KEGG, Pfam, Swiss-Prot, and eggNOG databases were compared. The functional annotation results are shown in Table 2. All 65,730 unigenes were annotated to the database, with an annotation ratio of 100%. Among them, 26,381 unigenes could be annotated to the GO database and accounted for 53.04%; 13,518 unigenes could be annotated to the KEGG database and accounted for 27.18%; 22,009 unigenes could be annotated to the Pfam database and accounted for 44.25%; 21,185 unigenes could be annotated to the Swiss-Prot database and accounted for 42.29%; 29,451 unigenes could be annotated to the eggNOG database and accounted for 59.21%; and 30,925 unigenes could be annotated to the non-redundant database and accounted for 62.17%. The quantitative comparison analysis revealed that the highest number of unigenes could be annotated to the non-redundant database, whereas the lowest number could be annotated to the KEGG database.

**TABLE 2:** Functional annotation results.

Annotated database	Number of notes	Note proportion (%)
All	65,730	100.00
GO	29,890	45.47
KEGG	23,835	36.26
Pfam	25,921	39.44
Swiss-Prot	24,274	36.93
eggNOG	33,657	51.20
NR	35,431	53.90

The non-redundant annotation results showed that 35,431 unigenes were annotated to the non-redundant database (Figure 4B). The most annotated species was carrot (*Daucus carota*) with a total of 15,497 unigenes (approximately 43.74%), which represented a source abundance of 38.17%. As for the other species, the homologous species of kiwifruit (*Actinidia chinensis*), grape (*Vitis vinifera*), coffee seed (*Coffea canephora*), and olive (*Olea europaea*) accounted for 7.67%, 5.37%, 1.77%, and 1.53% of the source abundance, respectively. Thus, the ginseng hairy-root tissue exhibited the greatest homology with carrots.

### Functional analysis and annotation of differentially expressed genes

To explore the molecular mechanisms underlying the response of ginseng hairy roots to ginsenoside Ro stress, a differential comparison analysis of the genes in the six transcriptome libraries was performed to obtain the differentially expressed genes (DEGs). The expression of the unigenes was calculated by transcripts per kilobase of exon model per million mapped reads. The Ro group was compared with the CK group (Ro vs. CK). The resulting differential genes between the treatment and control groups were defined as DEGs (*p* ≤ 0.05), which were analyzed using GO and KEGG enrichment analyses. As shown in Figures 4C,D 2,837 differentially expressed unigenes were identified in the Ro vs. CK comparison groups, including 1,380 upregulated and 1,457 downregulated unigenes. This indicated that the Ro treatment affected the expression of related genes in the ginseng hairy-root tissue and may be one of the main factors affecting the growth and development of ginseng hairy roots.

The results of the GO enrichment analysis of the DEGs are shown in Figure 4E. In the Ro vs. CK comparison groups, the DEGs were mainly involved in transcription, DNA-templated, transcriptional regulation, extracellular region, transcription factor activity, sequence-specific DNA binding activity, DNA binding transcription factor activity, DNA binding, response to oxidative stress, response to heat, multicellular organism development, and other processes. The results of the KEGG enrichment analysis of the DEGs are shown in Figure 4F. In the Ro vs. CK comparison groups, the DEGs were mainly involved in starch and sucrose metabolism, plant hormone signal transduction, plant–pathogen interaction, phenylpropanoid biosynthesis, glycerolipid metabolism, flavonoid biosynthesis, and other processes. The results indicated that phytohormone signal transduction played a greater role in the Ro treatment group.

The enrichment analyses of the DEGs revealed that the ginsenoside Ro treatment significantly affected the differential expression of the ginsenosides and genes regulating the hormone synthesis-related signaling pathway. The structures and biosynthesis of triterpenoid saponins are complex and diverse, and their key synthesis enzymes are rich; therefore, these compounds have received much attention in recent studies. Cytochrome P450 (CYP450) is an important enzyme involved in this process. As shown in Table 3, five genes related to CYP450 oxidase synthesis, including CYP82C4 and CYP87A3, were upregulated (TRINITY_DN15708_c0_g5 and TRINITY_DN25396_c0_g), and three genes were downregulated (TRINITY_DN22257_c0_g1, TRINITY_DN26346_c1_g1 and TRINITY_DN26346_c1_CK). The differential expression of the genes of key enzymes was observed in the plant hormone synthesis pathway, including three ARF7 and EFM genes (TRINITY_DN15428_c0_g1, TRINITY_DN15651_c0_g5, and TRINITY_DN22370_c0_g1) that were upregulated and two XTH23 and ZOX1 genes (TRINITY_DN13852_c0_g1 and TRINITY_DN15971_c0_g1) that were downregulated. This indicated that the Ro treatment regulated the expression of ginsenosides and hormone-related pathway genes.

**TABLE 3:** Differentially expressed genes related to ginsenosides and hormones.

Gene name	Gene ID	Swiss-Prot description	Ro TPM	CK TPM	Regulation
CYP82C4	TRINITY_DN22257_c0_g1	Cytochrome P450	37.32	78.79	Down
	TRINITY_DN26346_c1_g1	Cytochrome P450	35.87	88.65	Down
	TRINITY_DN15708_c0_g5	Cytochrome P450	30.80	12.26	Up
	TRINITY_DN26346_c1_g2	Cytochrome P450	16.17	36.10	Down
CYP87A3	TRINITY_DN25396_c0_g1	Cytochrome P450	19.22	9.48	Up
XTH23	TRINITY_DN13852_c0_g1	Plant hormone signal transduction	67.52	193.00	Down
	TRINITY_DN15428_c0_g1	Plant hormone signal transduction	79.97	39.10	Up
ARF7	TRINITY_DN15651_c0_g5	Plant hormone signal transduction	41.51	15.43	Up
EFM	TRINITY_DN22370_c0_g1	Plant hormone signal transduction	60.84	27.92	Up
ZOX1	TRINITY_DN15971_c0_g2	Zeatin biosynthesis	14.05	30.77	Down

To verify the reliability of the transcriptome data, 12 DEGs were randomly selected for a qPCR analysis to compare the expression levels between the samples. The results are shown in Figure 4G. The qPCR results were similar to the RNA-seq data, and the expression trends were consistent, which verified the reliability of the RNA-seq results.

### Combined mRNA and miRNA analysis

**FIGURE 5:**
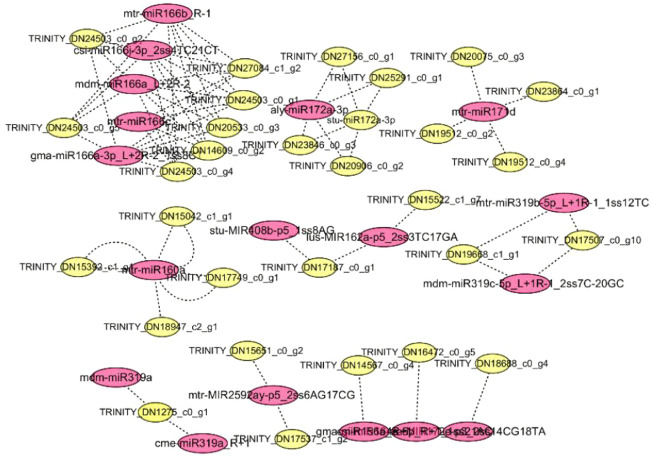
Networks of ginsenoside Ro-responsive miRNAs and their targets in ginseng. Red indicates miRNA; yellow indicates mRNA.

Using the ginseng transcriptome sequencing results as a reference, the differential miRNAs and predicted target genes were jointly analyzed, and a network diagram was constructed (Figure 5). miRNA plays a negative regulatory role. In the process of target gene prediction, one miRNA was often targeted to multiple target genes, such as mtr-miR171d and mtr-miR166b_R-1. Some miRNAs may have common target genes, such as aly-miR172a-3p and stu-miR172a-3p, which not only shows the diversity and specificity of miRNA target genes, but also indicates that miRNAs encoding the same target gene are involved in synergistic effects during plant growth and development. As shown in Figure 5, two known miRNAs (aly-miR172a-3p and mdm-miR166a_L+2R-2) had more predicted target genes, indicating that they play an important role in ginseng growth and the corresponding phytohormone signaling process. Through a combined analysis with mRNA, it was found that the target gene of ptc-miR156k_L+1, mtr-miR156b-5p, gma-miR156a_R+1, and mtr-miR156e was TRINITY_DN14567_c0_g4. TRINITY_DN14567_c0_g4 is a gene in the plant hormone signal transduction pathway. The mRNA sequencing analysis showed that the expression of the ZOX1 gene for zeatin synthesis was downregulated, the accumulation of ABA was inhibited, and the accumulation of ginsenosides decreased. The ZOX1 gene has an important relationship with ABA synthesis. Ginseng targets genes in the plant hormone signal transduction pathway through ptc-miR156k_L+1, mtr-miR156b-5p, gma-miR156a_R+1, and mtr-miR156e, thereby inhibiting their expression and reducing the expression of key enzyme genes in the hormone synthesis pathway. Their expression reduces the accumulation of phytohormones, which in turn affects the biosynthesis of ginsenosides. All four miRNAs were negatively correlated with mRNA, indicating that ptc-miR156k_L+1, mtr-miR156b-5p, gma-miR156a_R+1, and mtr-miR156e were most likely involved in the response to disorders relating to the continuous cropping of ginseng and the regulation of ginsenoside synthesis.

## Discussion

Terpenoids have always been important allelopathic substances and represent the main area of research on allelopathy. Ladhari identified two new dammarane-type triterpenoids and found that they inhibited seed germination and seedling growth in Amphora (Ladhari et al., 2020). The triterpenoid ginsenoside Rg1 has further been found to significantly inhibit the growth of Chinese cabbage seedlings (Hongrui et al., 2019). Furthermore, Yan *et al.* found that *P. notoginseng* releases saponins and other allelopathic substances into the soil through the decomposition of residues and root secretions (Yang et al., 2015). Plant roots secrete allelopathic substances into the soil to create a growth environment for pathogenic microorganisms and induce the growth of corresponding pathogenic microorganisms, thereby resulting in continuous cropping disorders (Adamczyk et al., 2021). *P. notoginseng* can release allelopathic substances such as phenolic acids and saponins into the soil through body decay and root secretion, which result in the death of root tissues (Yang et al., 2015). Ginsenoside Rg1 has further been found to significantly affect ginseng seed germination and seedling growth (Li and Lianxue, 2020). We found that the exogenous addition of different concentrations of ginsenoside Ro produced different degrees of the inhibition of the monthly multiplication rate of ginseng hairy roots. The Ro 0.5 group also exerted a significant inhibitory effect on the visual properties and viability of the ginseng hairy roots. The hairy roots gradually turned from white to dark yellow and brown and exhibited the worst growth state out of the treatment groups. All of the root-tip cells were stained red, which indicated that the cells had been damaged to a greater extent. Ginsenosides and other substances exert self-toxic effects on ginseng plants. Ginsenoside allelochemicals can degrade and shed ginseng fibrous root cells, and when the content of ginsenosides in ginseng plants decreases, the growth of ginseng is inhibited. These findings are consistent with those in this study (Bian et al., 2020; Wu et al., 2016; Zhan and Wang, 2021).

Phytohormones are organic compounds that are produced in plants that are usually transported from the synthesis site to the active site where they can regulate their physiological processes in trace amounts (Cong et al., 2016). Endogenous hormones play an important role in the regulation of physiological levels and gene expression when plants face changes in the external environment during their growth and development (Zhang et al., 2020). In this study, the amounts of the endogenous hormones IAA and SA in the hairy roots of ginseng increased significantly under Ro stress by 10.0% and 9.6%, respectively, compared with those in the CK group. However, the accumulation of ABA and its synthetic pathway intermediates was inhibited, as was the accumulation of the endogenous hormone JA and its synthetic pathway intermediates. IAA is a physiological response involved in identifying signals, inducing the expression of response genes, and regulating the corresponding physiological responses of the body during the early stages of plant growth and development (Qu et al., 2021; Zazimalova and Napier, 2003). SA plays an important role in plant growth and development, fruit ripening, and the stress response; however, environmental stress can promote SA accumulation in *Arabidopsis* and wheat (Cooper et al., 2018; Kosova et al., 2012). ABA regulates the production of secondary metabolites through a variety of factors, such as its synthesis pathway, signal transduction, and the interaction between hormones, whereas drought stress can induce a massive accumulation of ABA, thereby leading to stomatal closure and reduced water loss (Zhang et al., 2010,^2013^). JA and its methyl ester-based jasmonates comprise a class of plant growth regulators that play important roles in improving plant stress resistance and morphogenesis (Avanci et al., 2010). In this study, we speculated that ginseng hairy roots resist external environmental stimuli by regulating the biosynthesis of endogenous hormones. IAA, SA, JA, and ABA may be important endogenous signaling molecules in hairy ginseng roots that are involved in the response to stress induced by ginsenoside Ro.

In recent years, the application of transcriptomics to study medicinal plants has increased gradually. Medicinal plants that have undergone transcriptome sequencing include *P. ginseng* (Liu et al., 2016), *Panax quiquefolium* L*.* (Wu et al., 2013), *Salvia miltiorrhiza Bunge* (Gao et al., 2014), *Bupleurum chinense* (Sui et al., 2011), *Glycyrrhiza uralensis Fisch.* (Li et al., 2010), *Gastrodia elata Bl*. (Zeng et al., 2017), and *Dendrobium officinale Kimura et Migo.* (Shen et al., 2017). Through transcriptome sequencing, important progress has been made in the development of new gene mining, functional predictions, and metabolic pathways in medicinal plants. CYP450 is an ancient supergene family in plants. It partakes in a wide range of catalytic activities and is involved in plant biosynthesis and biological detoxification. CYP450 can catalyze the synthesis of compounds affecting plant growth and development, such as terpenoids, flavonoids, steroid alkaloids, alkaloids, and phenylpropanoids (Lee et al., 2010; Morikawa et al., 2006). Loss-of-function mutations in CYP72A69 can convert triterpenoid saponins from unwanted group A saponins to beneficial DDMP saponins (Yano et al., 2017). The CYP82 gene is moderately homologous to plant flavonoid hydroxylase, which is involved in the plant defense response and wound repair (Frank et al., 1996). In this study, we found that out of five genes (four copies of the CYP82C4 gene and one copy of the CYP87A3 gene) related to CYP450 oxidase synthesis, two genes were upregulated, and three were downregulated. The CYP82C4 and CYP87A3 genes may play an important role in ginsenoside Ro stress. Phytohormones play key regulatory roles in plant stress physiology and signal transduction in plant responses to biotic and abiotic stressors. The XTH23 gene can improve the resistance of plants to drought by increasing the amount of proline and superoxide dismutase and reducing the damage in plant cells under stress (Zhang et al., 2022). ARF and EFM are important transcription factors in plant hormone synthesis and play an important role in the plant response to environmental changes (Wu et al., 2011). In this study, we excavated the differential expression of four key enzyme genes in the plant hormone synthesis pathway. Three genes were upregulated, and one gene was downregulated. The XTH23, ARF, and EFM genes may further play an important role in ginsenoside Ro stress.

As the main means of regulation at the post-transcriptional level, miRNAs have become a popular topic in research on secondary metabolism in medicinal plants. miRNAs can regulate plant development and stress responses and have gradually become the focus of research on plant metabolic processes (Kidner and Martienssen, 2005; Owusu et al., 2021). Currently, 29 ginseng miRNAs have been published in the miRBase database (Wu et al., 2015). miRNA156 is one of the core members of the plant miRNA family and one of the most evolutionarily conserved miRNAs, which have been studied extensively in a variety of plants (Ma et al., 2021). miRNAs also play an important role in regulating the synthesis of plant secondary metabolites. The overexpression of miRNA156A can lead to increased levels of Dihydrofl avonol 4-reductase (DFR), which promotes anthocyanin biosynthesis (Gao et al., 2018). miRNA156 plays a role in *Arabidopsis* sesquiterpene biosynthesis and promotes sesquiterpene biosynthesis in the patchouli plant (Yu et al., 2015). miR156 targets 1-deoxy-D-xylose-5-phosphate synthase (1-deoxy-D-xylulose-5-phosphate synthase; DXS), which is involved in carotenoid synthesis in *Lycium barbarum* (Zeng et al., 2015). The mRNA sequencing analysis showed that the expression of the ZOX1 gene for zeatin synthesis was downregulated, the accumulation of ABA was inhibited, and the accumulation of ginsenosides decreased. The ZOX1 gene has an important relationship with ABA synthesis. The auxin-responsive factor ARF plays an important role in the response of plants to changes in the external environment, and JA and auxin are involved in the regulation of miRNA156 (Jerome et al., 2020). The upregulation of the ARF gene in ginseng in response to Ro stress affects the biosynthesis of the phytohormone JA, which is involved in the regulation of miRNA156. Using the results of the mRNA sequencing analysis as a reference, a joint analysis with mRNA was performed and revealed that the target gene of ptc-miR156k_L+1, mtr-miR156b-5p, gma-miR156a_R+1, and mtr-miR156e was TRINITY_DN14567_c0_g4, which is a gene in the plant hormone signal transduction pathway. Ginseng targets genes in the plant hormone signal transduction pathway through ptc-miR156k_L+1, mtr-miR156b-5p, gma-miR156a_R+1, and mtr-miR156e, thereby inhibiting their expression and reducing the expression of key enzyme genes in the hormone synthesis pathway. Their expression reduces the accumulation of phytohormones, which in turn affects the biosynthesis of ginsenosides.

## Conclusion

The results of this study indicate that the growth of ginseng hairy roots is significantly inhibited by Ro-induced stress (0.5 mg/L). The Ro stress also induced root-tip cell injury, which promoted the accumulation of the endogenous hormones IAA and SA and inhibited the accumulation of ABA and JA. Moreover, the accumulation of ginsenosides (except Rg3) was significantly inhibited under Ro stress. The mRNA analysis of the Ro (0.5 mg/L) and CK groups showed that the DEGs were mostly concentrated in the hormone signal transduction pathway. ARF7 and EFM were upregulated, whereas XTH23 and ZOX1 were downregulated, indicating that these genes are important candidates for hormone-responsive continuous cropping disorders. Furthermore, 74 differentially expressed miRNAs were identified from the miRNA sequencing analysis, of which 22 were upregulated and 52 were downregulated. The target gene of ptc-miR156k_L+1, mtr-miR156b-5p, gma-miR156a_R + 1, and mtr-miR156e was TRINITY_DN14567_c0_g4 in the plant hormone signal transduction pathway. These miRNAs were negatively correlated with mRNA, indicating their likely involvement in the ginseng response to continuous cropping disorders and the regulation of ginsenoside synthesis. Our findings provide a useful platform for overcoming the obstacles to the continuous cropping of ginseng and suggest new insights into the genetic engineering of plant stress responses.

## Materials and methods

### Plant materials and experimental design

Hairy roots have high genetic stability and fast reproduction rates. The hairy roots of *P. ginseng* were induced from the roots of ginseng plants (variety Erma Ya) that were obtained from the Key Laboratory of Chinese Medicine Planting and Development at the Changchun University of Chinese Medicine, Changchun, China. All the hairy roots were derived from the same ginseng root to ensure the uniformity of the test materials. The hairy roots of the ginseng group were selected as the experimental material.

The hairy roots of ginseng were first inoculated into 1/2 MS solid medium. After they had grown stably, 1 cm of the tips of the roots were transferred to liquid media. Six different concentrations of ginsenoside Ro aqueous solutions were prepared so that the final concentrations of the saponin Ro contained in the media were 0.002, 0.001, 0.05, 0.25, 0.5, and 1 mg/L. Distilled water was used for the control group. The control and treatment groups were set up with three biological replicates for each concentration dose and cultured continuously for 30 days. Hairy root samples were collected on day 30. A few of the samples were immediately frozen in liquid nitrogen and stored at −80°C for miRNA and transcriptome sequencing, and another sample was directly ground in a mortar and used for the extraction and analysis of ginsenosides.

### Determination of biomass

The growth of the hairy roots was measured using the weighing method. First, the liquid medium attached to the surface of the hairy roots was sucked dry, fresh weight was measured, and monthly multiplication ratio of the hairy roots was calculated. The calculation formula was as follows: monthly multiplication multiple = (harvest amount − inoculum amount)/inoculum amount × 100%. The morphological characteristics of the hairy roots were observed continuously for 30 days, and the shape, color, water content, and other data were recorded.

### Determination of cell viability

The hairy roots that had been cultured for 30 days were used to determine cell viability. The root tips of each treatment group were rinsed three times with deionized water and placed in a culture medium mixed with fluorescein diacetate and propidium iodide staining solution. They were then incubated for 40 min. The cells were stained in the dark and rinsed with normal saline for 5 min each to remove excess dye. Slides were mounted with an anti-fluorescence quenching mounting solution and placed under a laser confocal microscope for imaging. The excitation and detection wavelengths were 485 and 530 nm, respectively.

### Determination of ginsenoside content

The ginsenoside content in the ginseng hairy roots was detected using ultra-high-performance liquid chromatography tandem triple quadrupole mass spectrometry. The sample preparation method was as follows: A 1.1-g sample of the ginseng hairy roots was weighed accurately and placed into a conical flask containing 80 ml of 80% methanol, followed by ultrasonic oscillation for 0.5 h. This was repeated three times, and the supernatant was combined after centrifugation. Two supernatants were combined, concentrated under reduced pressure on a rotary evaporator to remove the methanol, and extracted with chloroform three times. The chloroform layer was then discarded, and the supernatants were again extracted three times with saturated n-butanol and water. The n-butanol layer was then collected and combined. Finally, the supernatant was subjected to a nitrogen blow-dry, diluted to 1 ml with 80% chromatographic methanol, filtered through a 0.22-μm filter membrane, and stored at 4°C for later use.

The liquid chromatography separation conditions were as follows: Thermo C18 column (50 mm × 3 mm, 1.7 μm), mobile phase with 0.1% formic acid (A)-acetonitrile (B), gradient elution for 0–5 min, 19% B; 5–29 min, 19%–25% B; 29–72 min, 25%–40% B; 72–77 min, 40%–90% B; 77–80 min, 90% B; 80–83 min, 90%–19% B; 83–88 min, 19% B; flow rate of 0.2 ml/min, column temperature of 35°C, sample chamber temperature of 4°C, and injection volume of 5 μl. The mass spectrometry detection conditions were as follows: electrospray ionization, negative ion mode, and multiple reaction monitoring; mass scanning range m/z 100–1,500; spray voltage: 2,500 V; sheath gas pressure: 35 arb; auxiliary gas pressure: 10 arb; transfer capillary temperature: 350°C; nebulizer temperature: 300°C. Notoginsenoside R1, Rg1, Re, Rf, Rg2, Rh1(S), F3, Rb1, Rc, Ro, Rb2, Rd, F2, and Rg3 standards were used to establish a standard curve as shown in Supplementary Table S4. The extracted ion chromatogram is shown in Supplementary Figure S1.

### Determination of the endogenous hormone content

The endogenous hormone content in the hairy roots of the ginseng was determined using liquid chromatography with tandem mass spectrometry. The hairy root samples were pre-frozen in an ultra-low-temperature freezer then ground using a grinder at a speed of 30 Hz for 1 min to form a powder. The powder (50 mg) was weighed, an appropriate amount of the internal standard was added, and 1 ml of methanol/water/formic acid (15:4:1) was used as the extraction solvent. The extract was concentrated, reconstituted with 100 μl of an 80% methanol/water solution, passed through a 0.22-μm filter membrane, and placed in a liquid phase bottle for mass spectrometry. The liquid chromatography conditions were as follows: Waters ACQUITY UPLC HSS T3 C18 column (1.8 µm, 100 mm × 2.1 mm). Mobile phase A: 0.04% acetic acid in water; phase B: acetonitrile (0.04% acetic acid). Gradient elution program: 0–1.0 min A/B 95:5, 1.0–8.0 min A/B 95:5, 8.0–9.0 min A/B 5:95, 9.0–9.1 min 5:95, 9.1–12.0 min 95:5, 12.0 min 95:5. The flow rate was 0.35 ml/min, column temperature was 40°C, and injection volume was 2 μl. The mass spectrometry conditions were as follows: electrospray ionization temperature of 550°C, mass spectrometry voltages of 5,500 and −4,500 V in positive and negative ion modes, respectively, and a curtain gas pressure of 35 psi. In the Q-Trap 6500+, each ion pair was scanned according to the optimized declustering potential and collision energy. All of the samples were measured three times, and the average values were compared.

### mRNA library construction and sequencing

Total RNA was extracted from the ginseng hairy-root tissue samples according to the manufacturer’s instructions for the TRIzol reagent (Invitrogen, CA, United States) and purified using DNase. We used a NanoDrop 2000 nucleic acid UV spectrophotometer to measure the RNA sample purity at OD 260/280 between 1.8 and 2.2. An Agilent Bioanalyzer 2100 was used to measure the total RNA amount and purity (25 S:18 S ≥ 1.0; total RNA ≥2 μg; RIN value >7.0). Qualified RNA was used to construct an RNA library. Poly-A-tailed mRNA was enriched from the total RNA using magnetic beads with oligo (dT). By adding a fragmentation reagent fragmentation buffer, the extracted mRNA was randomly fragmented into small fragments of approximately 200 bp. Using the fragmented mRNA as a template, one-strand cDNA was synthesized with six-base random hexamers, followed by the addition of buffer, dNTPs, RNase H, and DNA polymerase I to synthesize two-strand cDNA to form a stable double strand. The double-stranded product was purified using AMPure XP Roads, and the DNA cohesive end was repaired to blunt ends using T4 DNA polymerase and Klenow DNA polymerase, followed by the ligation of base A at the 3′ end to ligate the Y-linker. Fragment selection was performed using AMPureXP Roads, and the sorted products were used for PCR amplification and purification to obtain the final library. After the library had been qualified, the Illumina HiSeq 4000 system at LC-BIO (Hangzhou, China) was used for sequencing. The sequencing read length was 2 bp × 150 bp (double-ended) (PE150).

### *De novo* assembly, unigene annotation, functional classification, and differential expression analysis

Cutadapt and Perl scripts were used in-house to remove the reads that contained adaptor contamination, low-quality bases, and undetermined bases (Martin, 2011). Sequence quality was assessed using FastQC (http://www.bioinformatics.babraham.ac.uk/projects/fastqc/) and the Q20, Q30, and GC content of the clean data. All of the downstream analyses were based on the high-quality clean data. The *de novo* assembly of the transcriptome was performed using Trinity 2.4.0 (Grabherr et al., 2011), which groups transcripts into clusters based on the shared sequence content. Such transcript clusters are loosely referred to as “genes.” The longest transcript in the cluster was chosen as the “gene” sequence, or unigene. All of the assembled unigenes were aligned against the non-redundant protein database (http://www.ncbi.nlm.nih.gov/) and the GO (http://www.geneontology.org), Swiss-Prot (http://www.expasy.ch/sprot/), KEGG (http://www.genome.jp/kegg/), and eggnog (http://eggnogdb.embl.de/) databases using DIAMOND (Buchfink et al., 2015) with a threshold of Evalue <0.00001. The gene and unigene expression levels (in transcripts per kilobase of exon model per million mapped reads) were quantitatively analyzed using Salmon software. After obtaining the number of gene sequences, the R package edgeR was used to analyze the differences in gene expression between the samples using log2 (fold change) > 1 or log2 (fold change) < −1 as the screening standard (*p* < 0.05). The gene expression was analyzed, and the functions and pathways in which the DEGs were enriched were assessed using GO and the KEGG (Mortazavi et al., 2008; Patro et al., 2017; Robinson et al., 2010).

### miRNA library construction and sequencing

The TruSeq Small RNA Sample Prep Kit (Illumina, San Diego, CA, United States) was used to prepare the miRNA sequencing library. We obtained small RNA fragments (15–50 nt) and mixed the total RNA with an equal volume of 2 × loading buffer mix and incubated the mixture at 65°C for 5 min to eliminate the interference of secondary structures in the RNA. We then used 15% TBE polyacrylamide gel electrophoresis (TBE PAGE) for 15 min to separate the total RNA bands and perform further cutting and purification. This allowed us to obtain small RNA fragments with a length of 15–50 nt. T4 RNA ligase 2 from NEB Company (United States) was used to connect the 3′-end adapter to the small RNA fragments. TBE PAGE (15%) was then used for 15 min to separate the RNA bands, which were further cut and purified to obtain small RNA fragments with a length of 41–76 nt. T4 RNA ligase 2 from NEB Company was further used to connect the 5′-end adapter with the small RNA fragments, after which 15% TBE PAGE was used for 15 min to separate the RNA bands, which were further cut and purified to obtain small RNA fragments with a length of 64–99 nt. For the reverse transcription reaction and PCR amplification, we used the SuperScript II Reverse Transcriptase system according to the manufacturer’s instructions with incubation in a preheated thermal cycler at 50°C for 1 h to obtain reverse-transcribed single-stranded cDNA. DNA polymerase and amplification primers were cyclically amplified to obtain a cDNA library. To purify the small RNA library, we performed 6% Novex TBE PAGE for 1 h at 145 V and cut the fragment region to a length of 80–115 bp. After the obtained fragment had been eluted and purified, the cDNA was further quantified using a NanoDrop nucleic acid and protein concentration analyzer. The quality of the library was checked using Agilent 2100 and qPCR. The Illumina HiSeq 2500 was used to sequence the qualified library. The sequencing read length was 1 bp × 50 bp (single-ended).

### Analysis of the differentially expressed miRNAs and target gene prediction

The differential expression of the miRNAs based on normalized deep-sequencing counts was analyzed selectively using Fisher’s exact test, the Chi-squared 2 × 2 test, Chi-squared nXn test, Student’s *t*-test, or an analysis of variance based on the experimental design. The significance threshold was set to 0.01 and 0.05 in each test. To predict the genes targeted by the miRNAs, computational target prediction algorithms (PsRobot, v1.2) were used to identify the miRNA binding sites. The GO terms and KEGG pathways of the most abundant miRNA targets were also annotated.

### Verification by qRT-PCR

To verify the reliability of the obtained transcriptome and miRNA data, RT-PCR was performed on 13 randomly selected DEGs in the transcriptome data and 10 differentially expressed miRNAs with different expression levels. Using the actin gene as an internal reference, primers were designed with Primer 5.0 software and synthesized by Shanghai Sangon Bioengineering Co., Ltd. The primer sequences are shown in Supplementary Tables S5, S6.

## Data Availability

The datasets presented in this study can be found in online repositories. The names of the repository/repositories and accession number(s) can be found in the article/Supplementary Material.

## Abbreviations^1^

ABA, abscisic acid; ANOVA, analysis of variance; IAA, indole-3-acetic acid; JA, jasmonate acid; POD, peroxidase; SA, salicylic acid; SOD, superoxide dismutase; UHPLC-QQQ-MS, ultra-high performance liquid chromatography tandem triple quadrupole mass spectrometry.
