# Supplementary figures and images for: Regulatory network of ginsenoside biosynthesis under Ro stress in the hairy roots of Panax ginseng revealed by RNA sequencing
Source: Front Bioeng Biotechnol. 2022 Oct 31;10:1006386. doi: 10.3389/fbioe.2022.1006386 (PMC9659575; doi:10.3389/fbioe.2022.1006386)

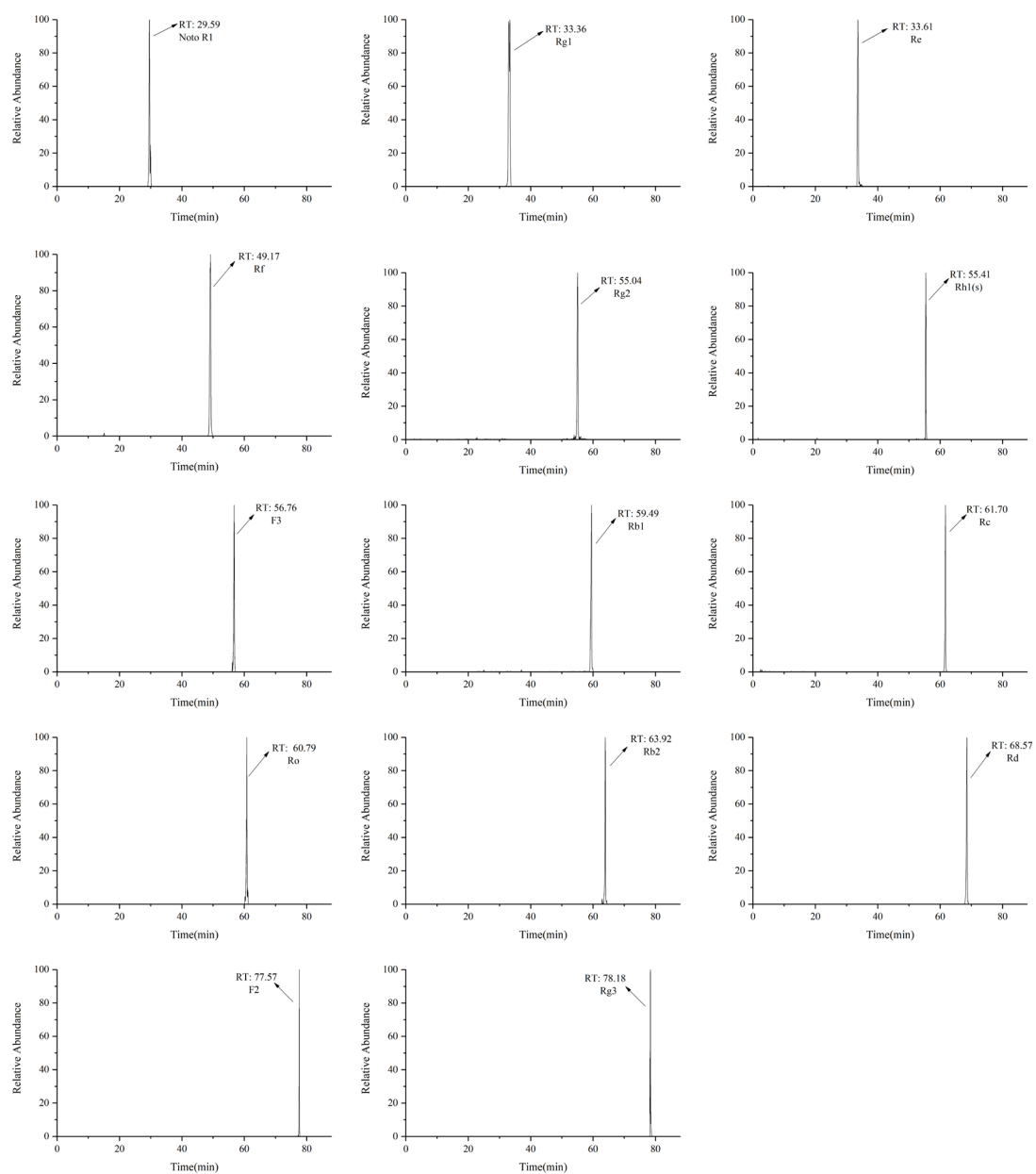

**Fig. S1.** Ion flow diagrams of 14 ginsenosides extracted from the hairy roots of ginseng.

Supplement: Supplementary file 1 [file Image1.pdf]
